# Supplementary material for: Cross-platform motif discovery and benchmarking to explore binding specificities of poorly studied human transcription factors
Source: Commun Biol. 2025 Nov 7;8:1545. doi: 10.1038/s42003-025-08909-9 (PMC12594988; doi:10.1038/s42003-025-08909-9)
Supplement: Supplementary file 2 — Supplementary Information [file 42003_2025_8909_MOESM2_ESM.pdf]

Cross-platform motif discovery and benchmarking to explore binding specificities of poorly studied human transcription factors

Supplementary Information

Ilya E. Vorontsov, Ivan Kozin, Sergey Abramov, Alexandr Boytsov, Arttu Jolma, Mihai Albu, Giovanna Ambrosini, Katerina Faltejskova, Antoni J. Gralak, Nikita Gryzunov, Sachi Inukai, Semyon Kolmykov, Pavel Kravchenko, Judith F. Kribelbauer-Swietek, Kaitlin U. Laverty, Vladimir Nozdrin, Zain M. Patel, Dmitry Penzar, Marie-Luise Plescher, Sara E. Pour, Rozita Razavi, Ally W.H. Yang, Ivan Yevshin, Arsenii Zinkevich, Matthew T. Weirauch, Philipp Bucher, Bart Deplancke, Oriol Fornes, Jan Grau, Ivo Grosse, Fedor A. Kolpakov, The Codebook/GRECO-BIT Consortium,
Vsevolod J. Makeev, Timothy R. Hughes, Ivan V. Kulakovskiy

Contents (pdf): Supplementary Figures 1-12.

Supplementary Data (xlsx):

Supplementary Data 1. Overview of the Codebook experiments used in the study.

Supplementary Data 2. Analysis of the transferability of Archipelago models.

Supplementary Data 3. List of software tools used in the study.

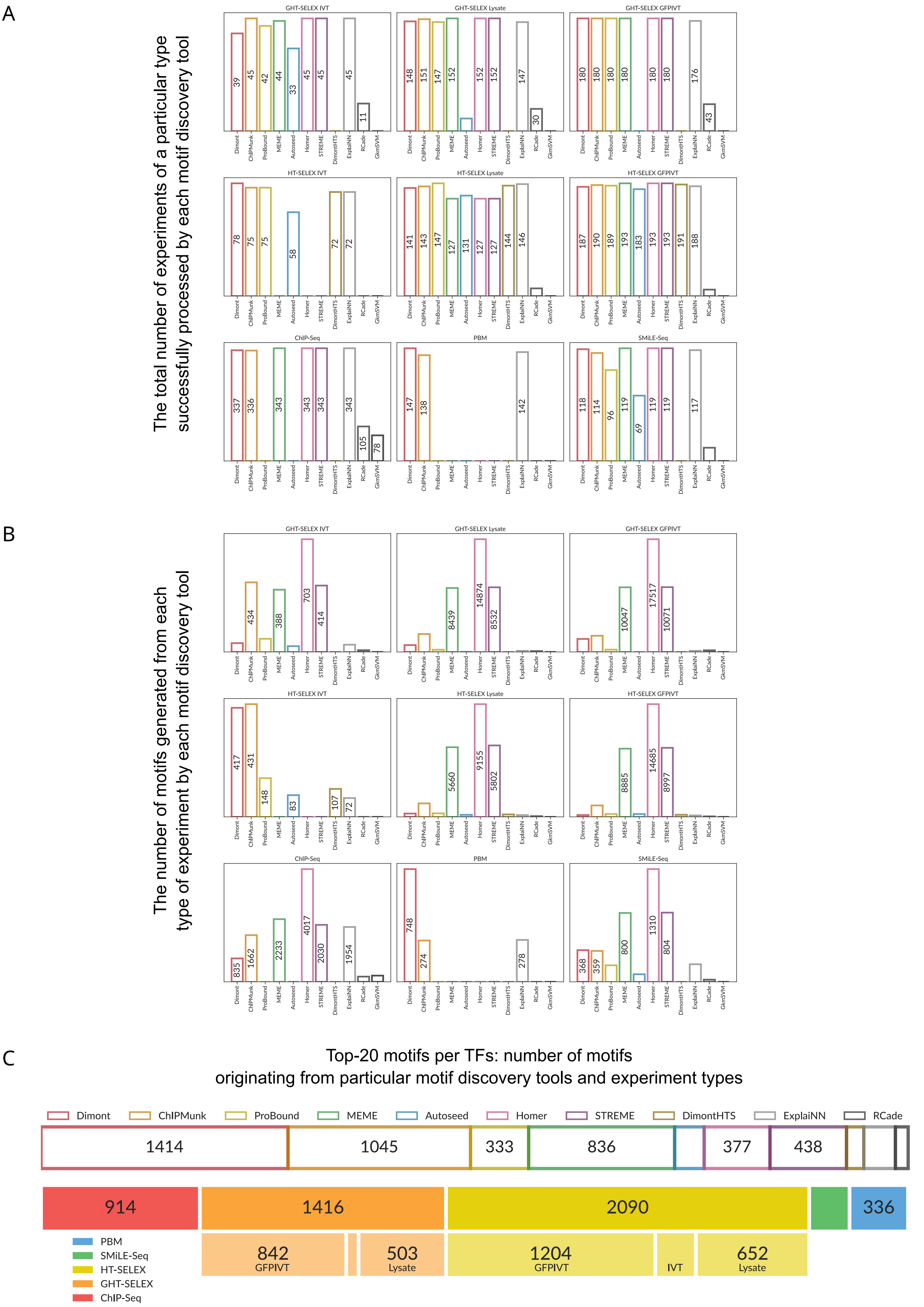

Supplementary Figure 1. A: The number of experiments successfully processed by each motif discovery tool. **B:** The number of motifs generated from each type of experiment by each motif discovery tool. **C:** The composition of the collection of overall top-20 motifs shown in the same way as the set of top-ranking motifs in Figure 1.

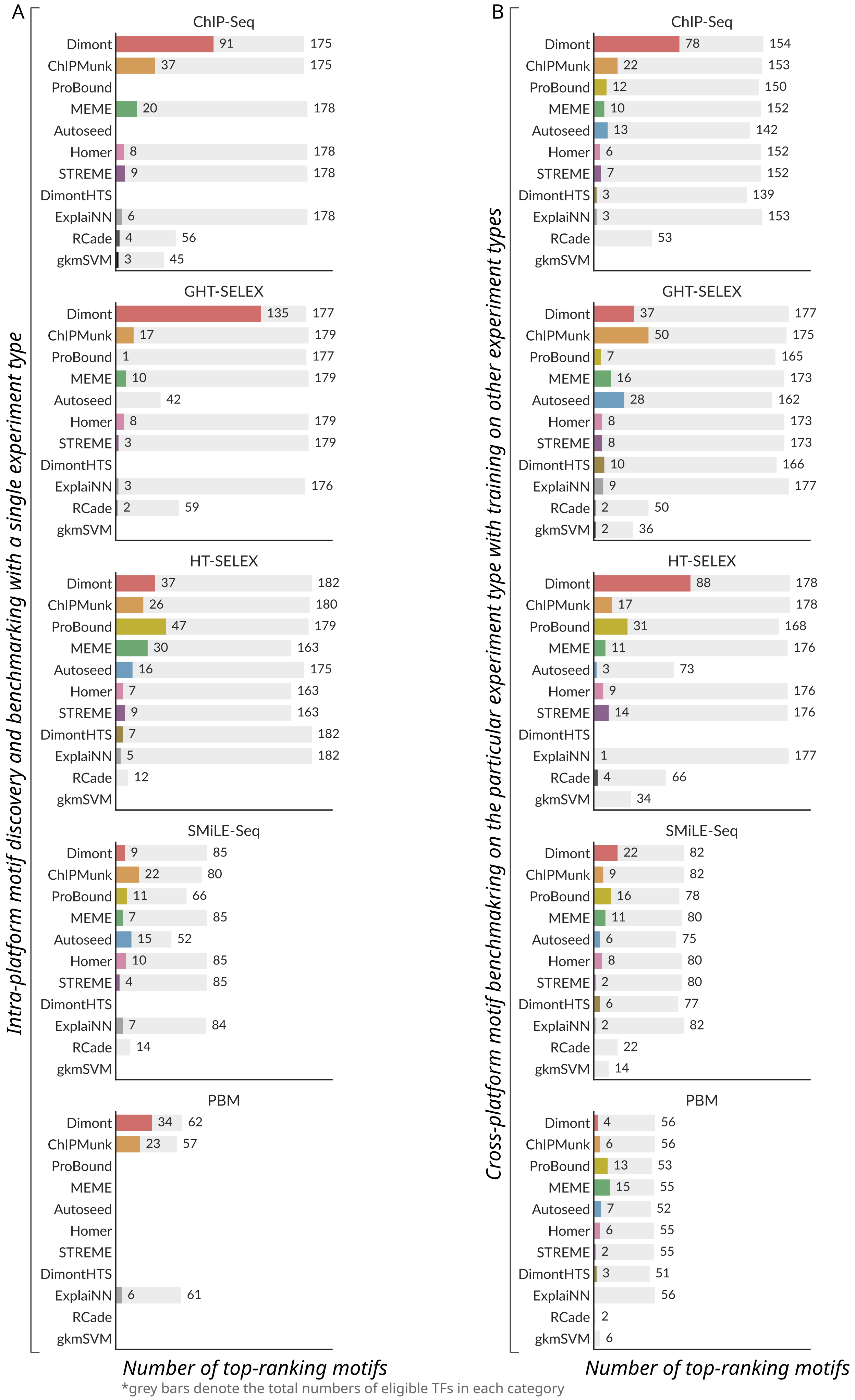

Supplementary Figure 2. Numbers of top-ranking motifs yielded by different motif discovery tools detected in intra- (single experiment type) and cross-platform benchmarking (between experiment types). **A:** The number of motifs (TFs) for which a particular tool produced the top-ranking motif if trained and tested on the same type of experiment. **B:** The number of motifs (TFs) for which a particular tool yielded the top-ranking motif when tested on a particular type of experiment but trained on any of the other types. The total number of TFs with at least one benchmarked motif for a particular combination of a tool and an experiment type is shown in gray.

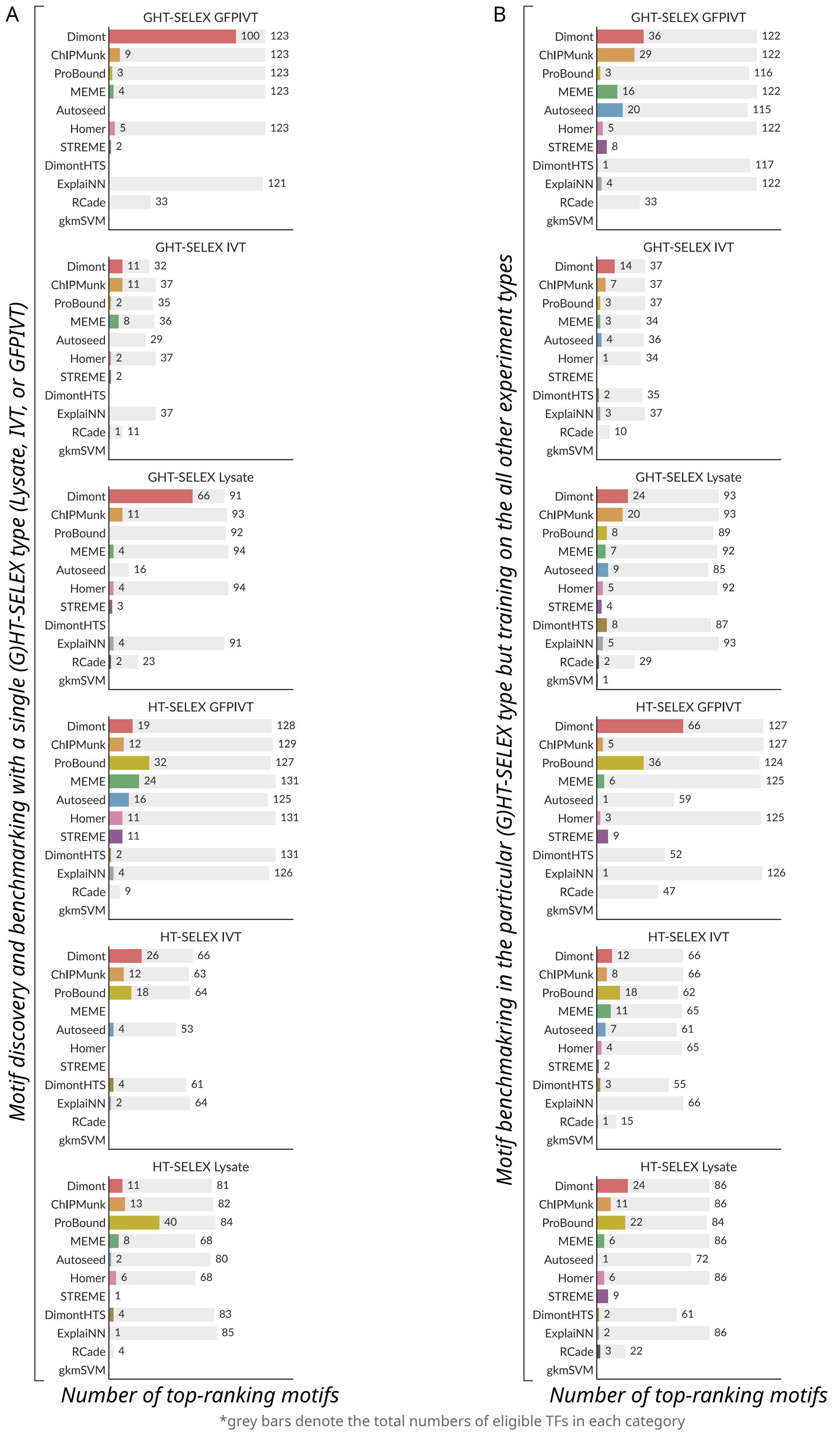

Supplementary Figure 3. Numbers of top-ranking motifs from different motif discovery tools detected in intra- and cross-platform benchmarking considering types of GHT-SELEX and HT-SELEX (IVT, Lysate, GFPIVT) independently. **A:** Motif discovery and testing performed with the same experiment type. **B:** Motif discovery and testing performed with different platforms, including training and testing between (G)HT-SELEX types.

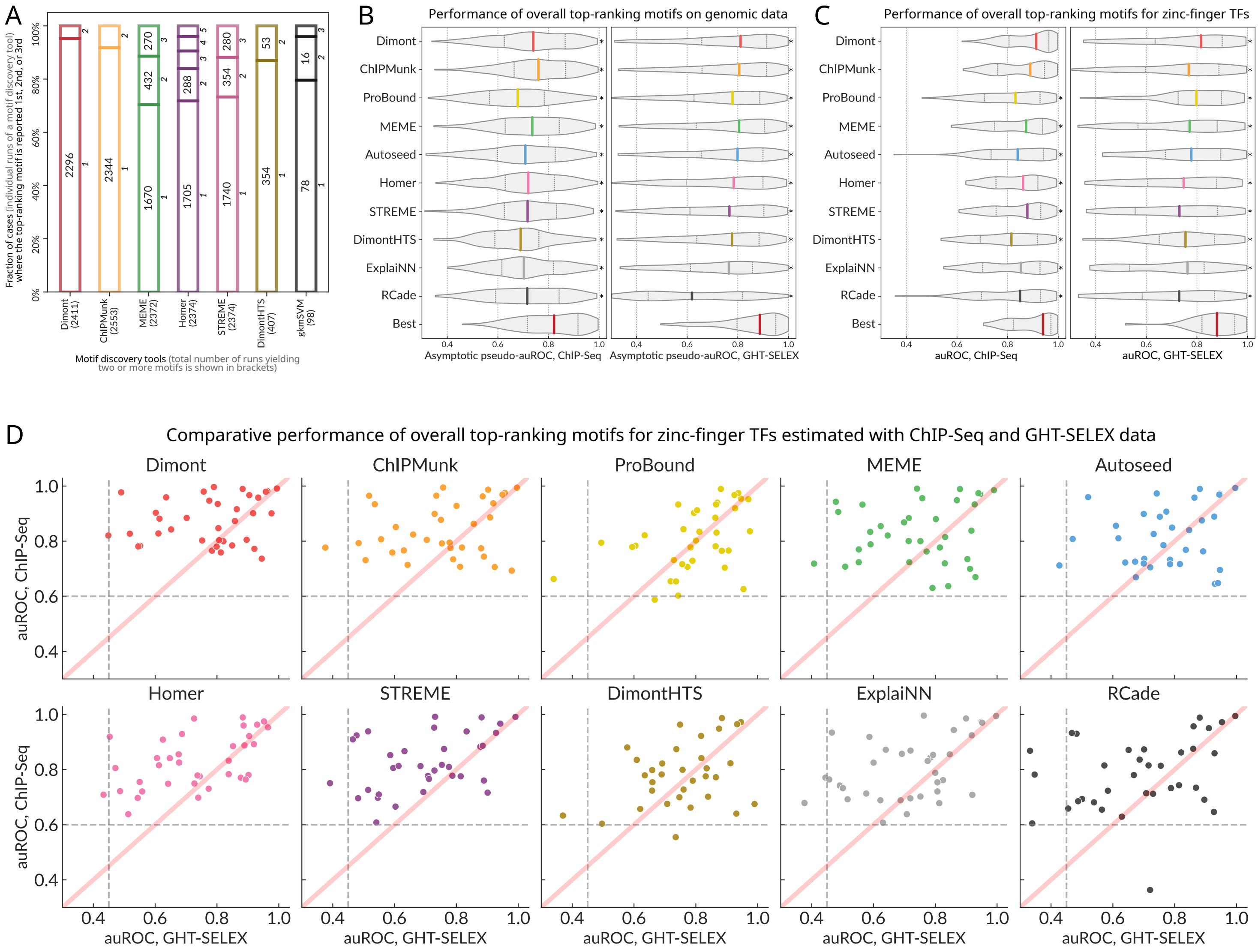

Supplementary Figure 4. A. The fraction of cases where the 1st, the 2nd, or the 3rd motif reported by a single run of a motif discovery tool are ranked the highest of these three in the overall benchmarking. **B:** Performance achieved by the overall top-ranking motifs produced by individual tools across TFs: distributions of pseudo-auROC values for ChIP-Seq and GHT-SELEX. **C:** Distributions of auROC values for ChIP-Seq and GHT-SELEX for zinc-finger TFs only. **D:** Scatterplots of auROC achieved at GHT-SELEX and ChIP-Seq for zinc-finger TFs only. *$p < 0.05$, paired Wilcoxon test against the best of motifs from all listed tools.

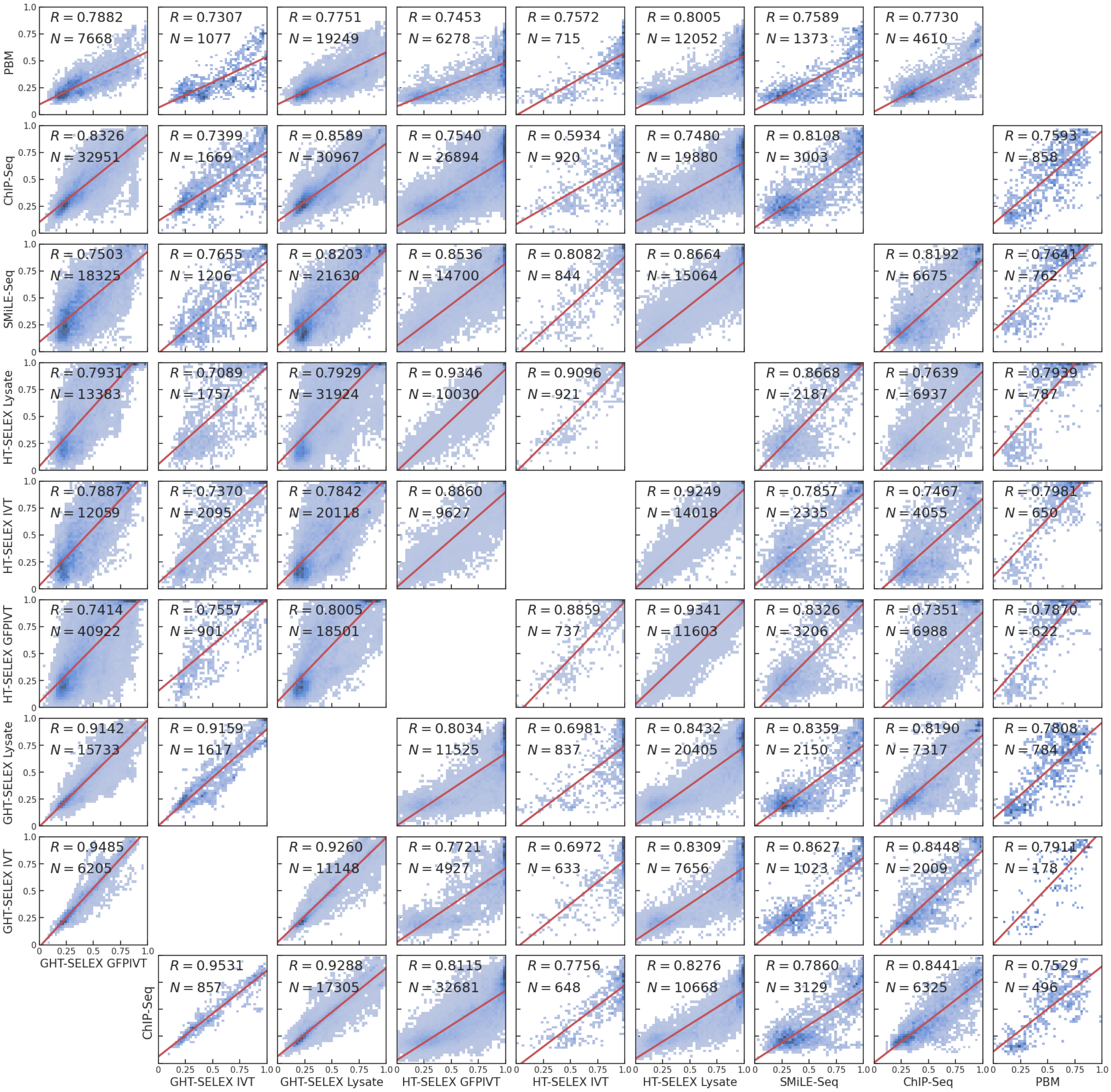

Supplementary Figure 5. Correlation between overall performance of motifs at the data of the same type as used for motif derivation (intra-platform) and at the test data from other experiment types (cross-platform).

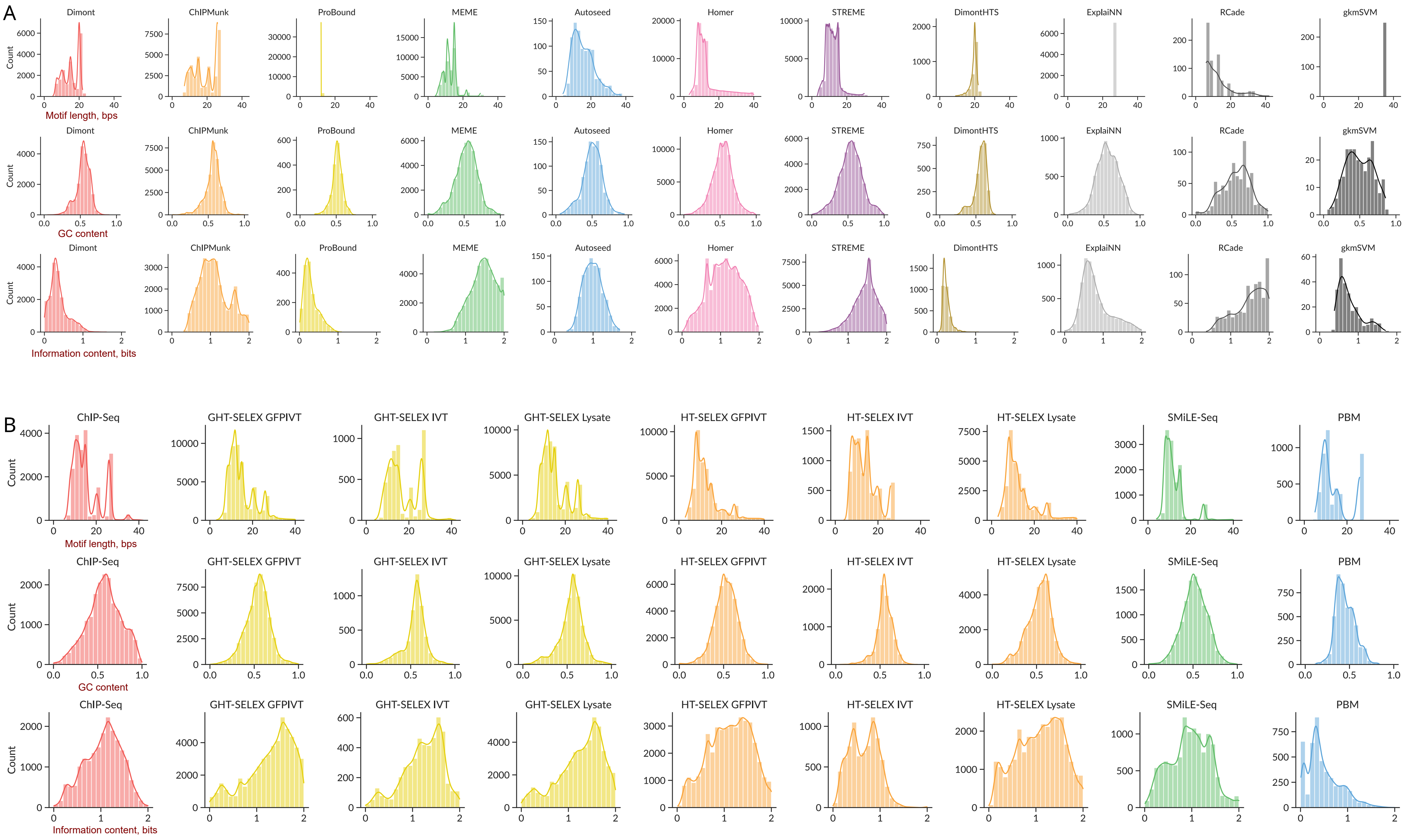

Supplementary Figure 6. Distributions of basic motif properties (top to bottom: length, GC%, information content).
A: Different motif discovery tools. **B:** Different experiment types.

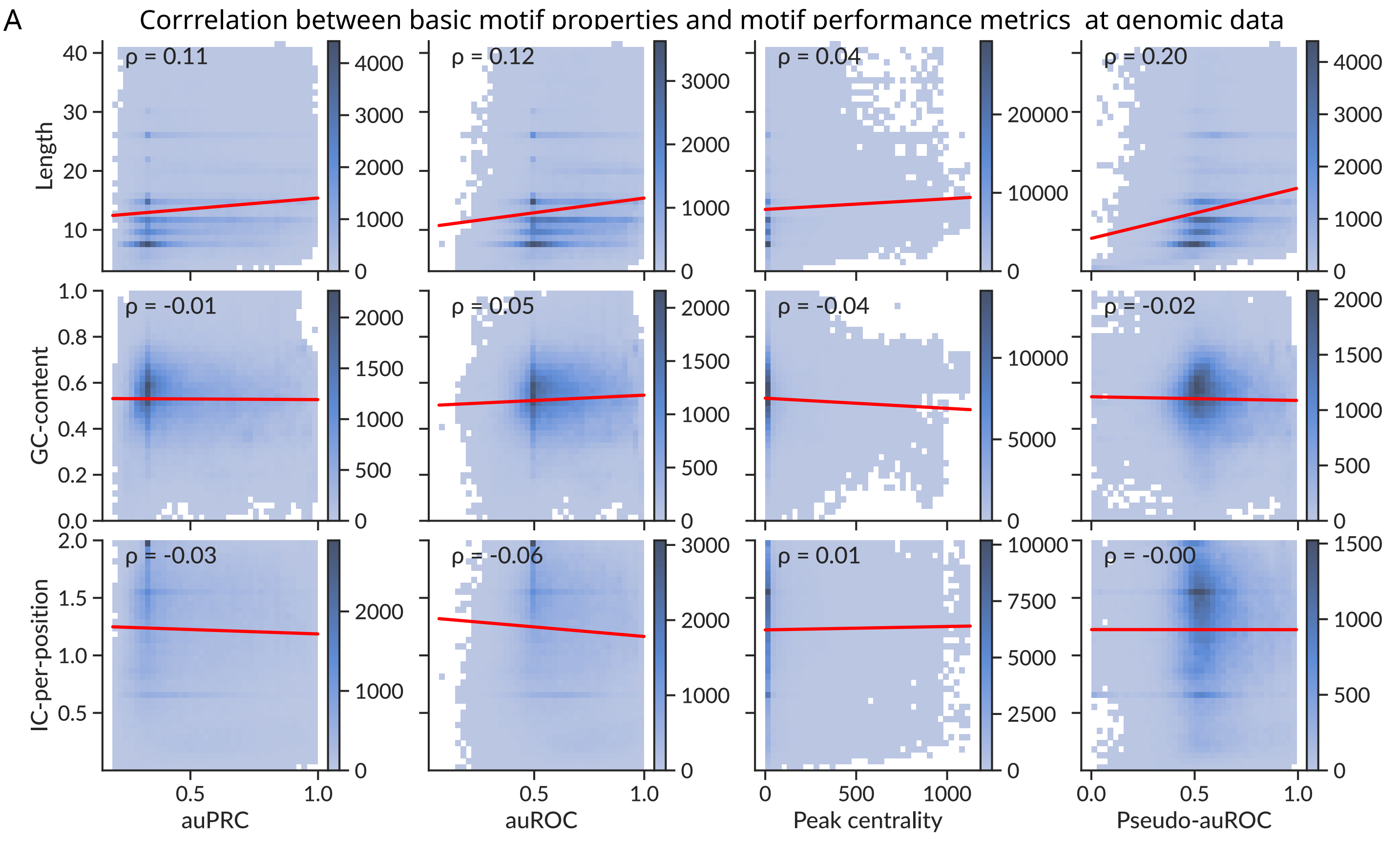

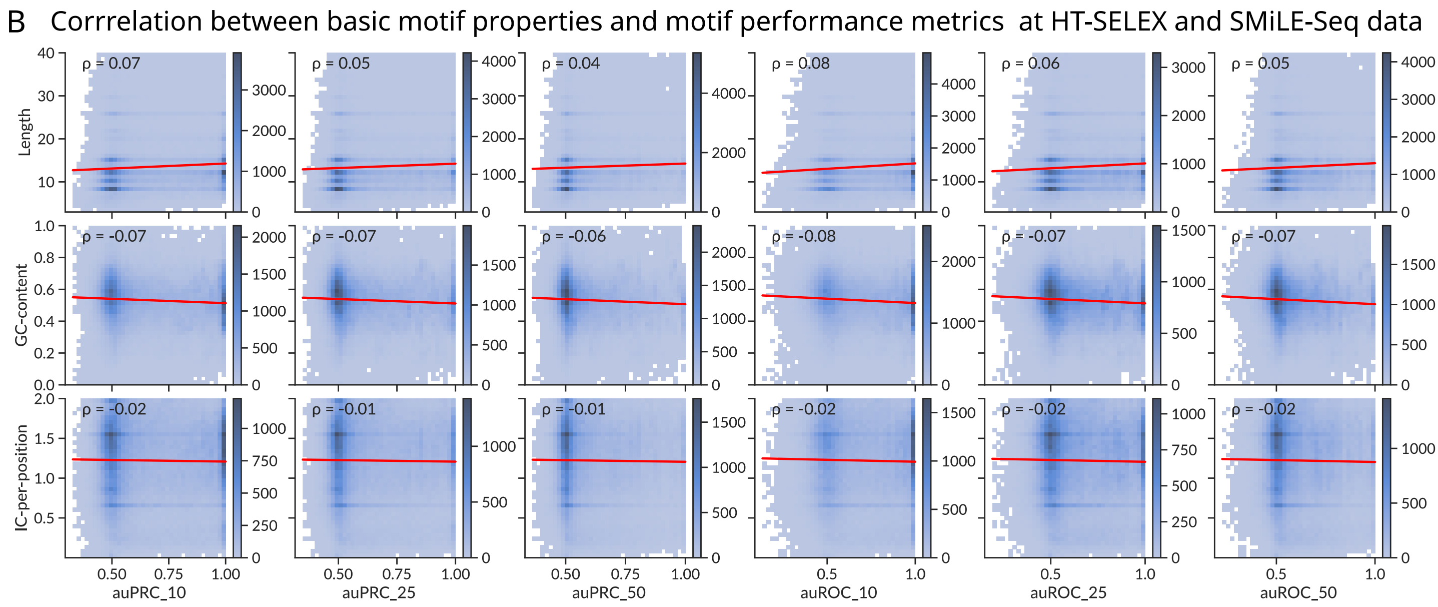

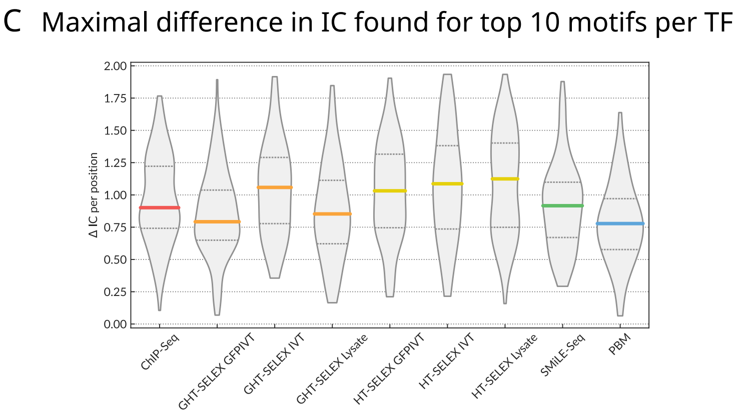

Supplementary Figure 7. A: Correlations between individual performance metrics and basic motif properties (length, information content, GC%) for ChIP-Seq and GHT-SELEX test data. The color scale of the binned density plots denotes the numbers of motifs. **B:** Same as (A) for benchmarks with artificial sequences from HT-SELEX and SMiLE-Seq. **C:** A violin plot across TFs illustrating the maximal difference in the information content for the top 10 motifs for each TF. Individual experiment types are shown separately.

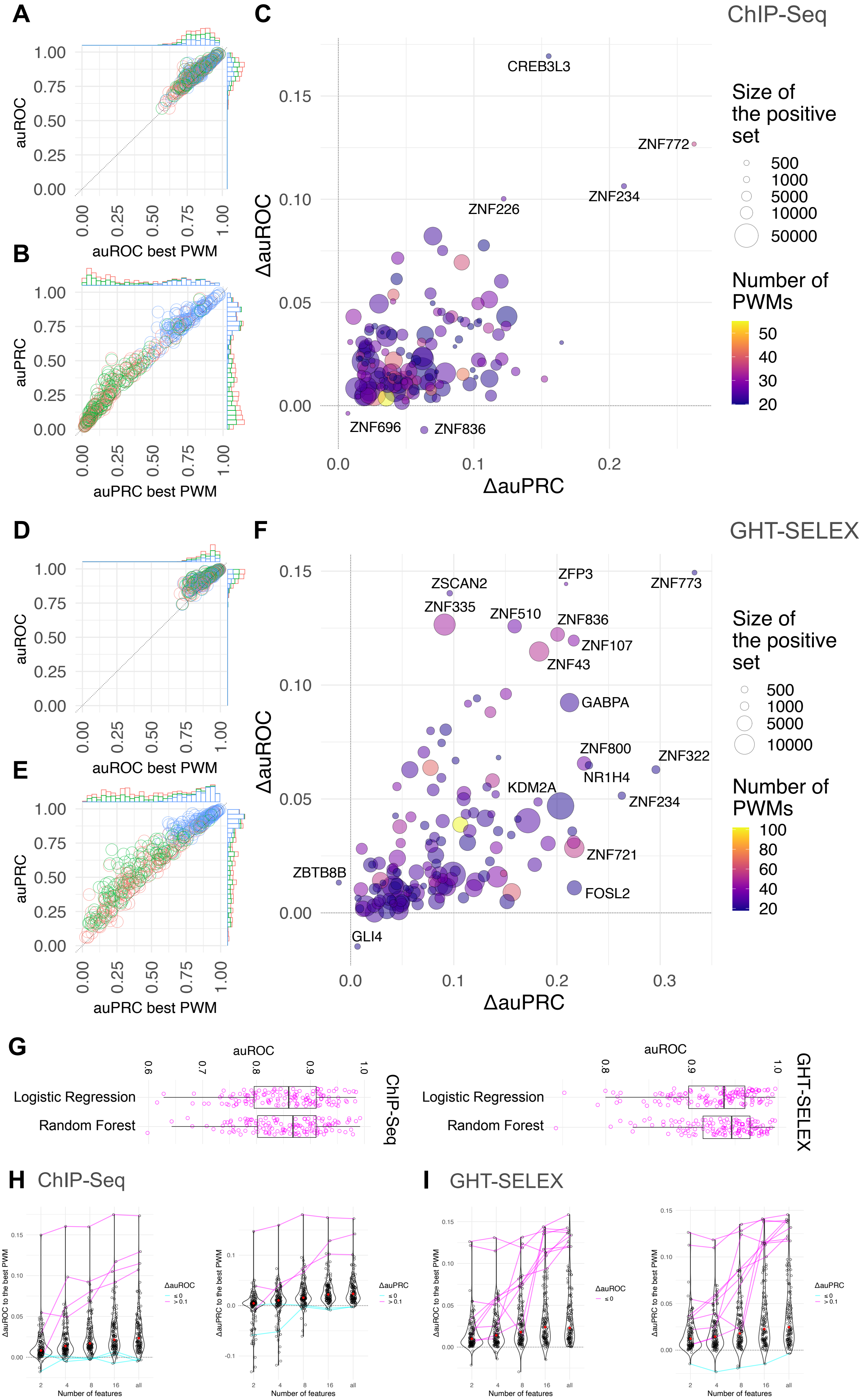

Supplementary Figure 8. Performance of the Archipelago model when trained and tested on the same type of experimental data. **A-C:** Absolute performance and relative gain of the Archipelago model compared to the best PWMs, ChIP-Seq. **D-F:** Same as **A-C** but for GHT-SELEX. Estimates obtained with alternative negative sets (random, aliens, and shades) are depicted by colored circles (green, red, and blue, respectively). **G:** Performance of the Random Forest and Logistic regression achieved when trained and tested on ChIP-Seq or GHT-SELEX data. **H, I:** Performance gain (purple) or loss (blue) depending on the number of PWMs in the model, ChIP-Seq and GHT-SELEX data.

CREB3L3

ChIP-Seq		GHT-SELEX	
1	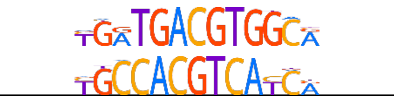	1	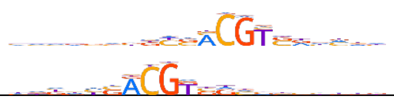
2	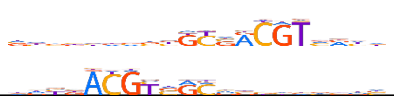	2	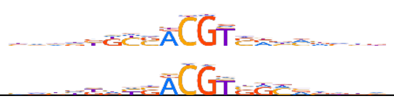
3	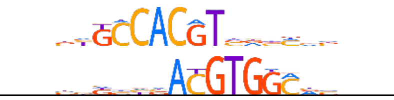	3	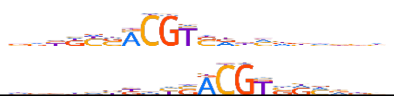
4	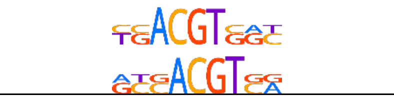	4	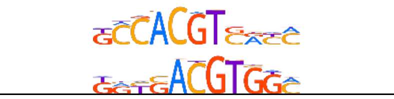
5	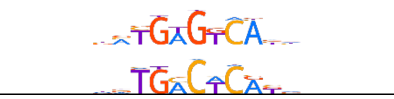	5	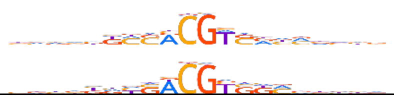

FOSL2

ChIP-Seq		GHT-SELEX	
1	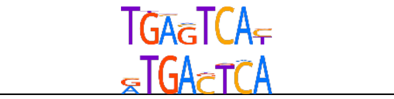	1	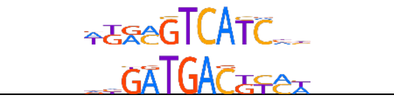
2	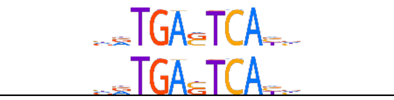	2	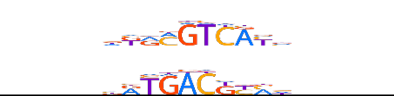
3	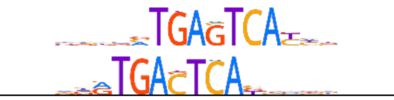	3	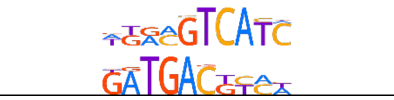
4	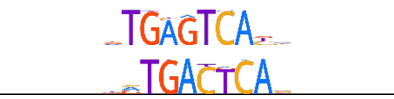	4	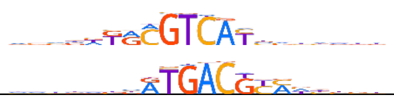
5	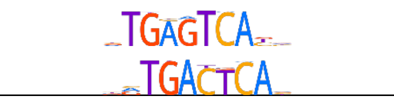	5	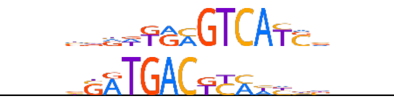

Supplementary Figure 9. Top 5 motifs of the Archipelago models sorted by feature importance. Each row lists the motif in both possible orientations.

ZNF772

	ChIP-Seq		GHT-SELEX
1	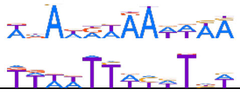	1	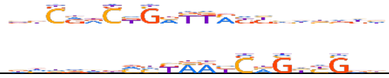
2	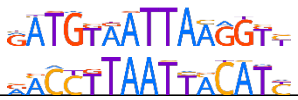	2	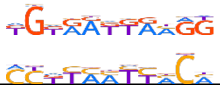
3	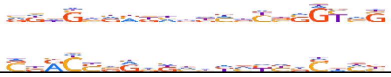	3	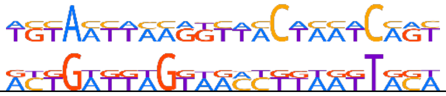
4	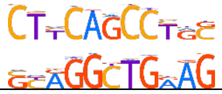	4	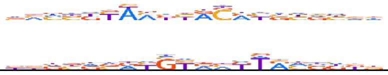
5	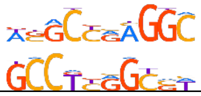	5	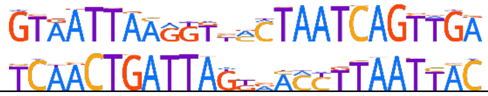

ZNF773

	ChIP-Seq		GHT-SELEX
1	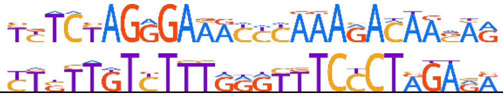	1	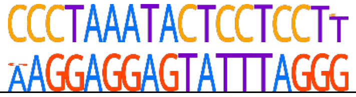
2	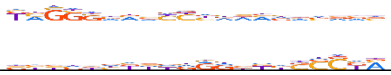	2	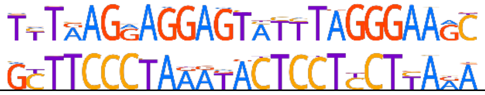
3	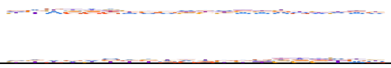	3	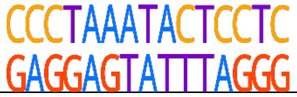
4	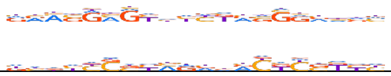	4	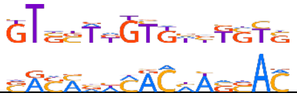
5	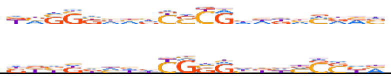	5	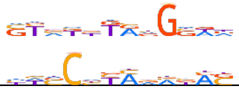

Supplementary Figure 9, cont.

ZNF770

ChIP-Seq		GHT-SELEX	
1	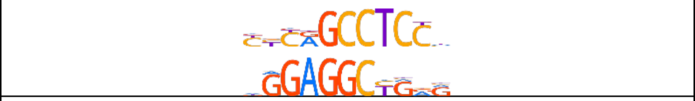	1	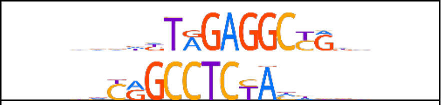
2	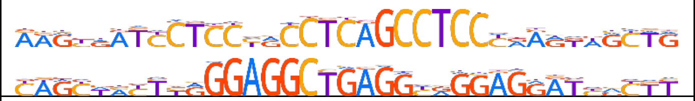	2	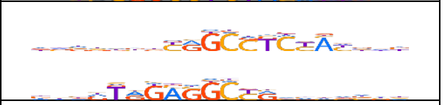
3	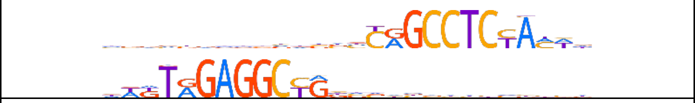	3	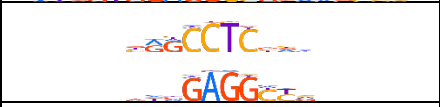
4	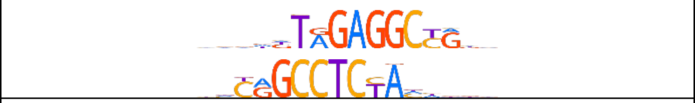	4	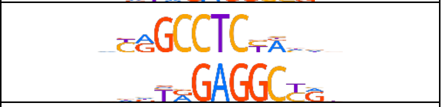
5	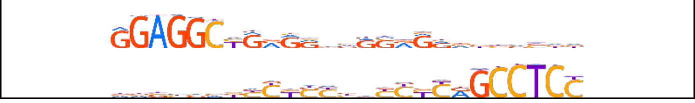	5	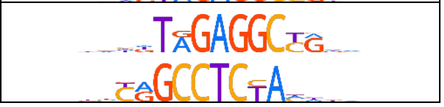

ZNF43

ChIP-Seq		GHT-SELEX	
1	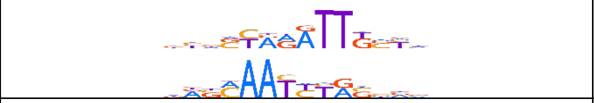	1	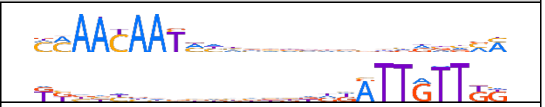
2	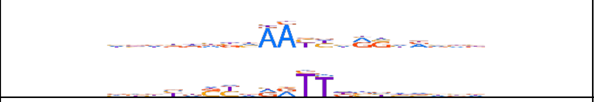	2	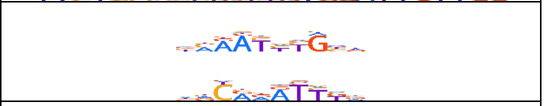
3	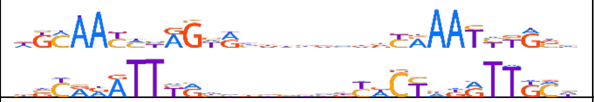	3	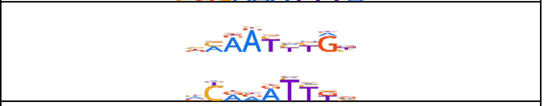
4	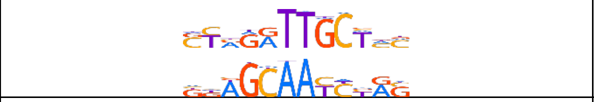	4	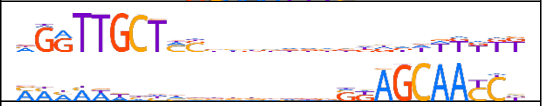
5	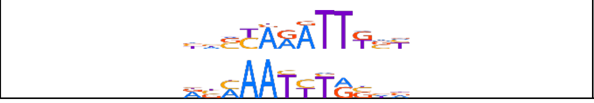	5	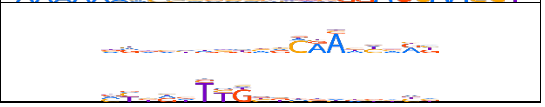

Supplementary Figure 9, cont.

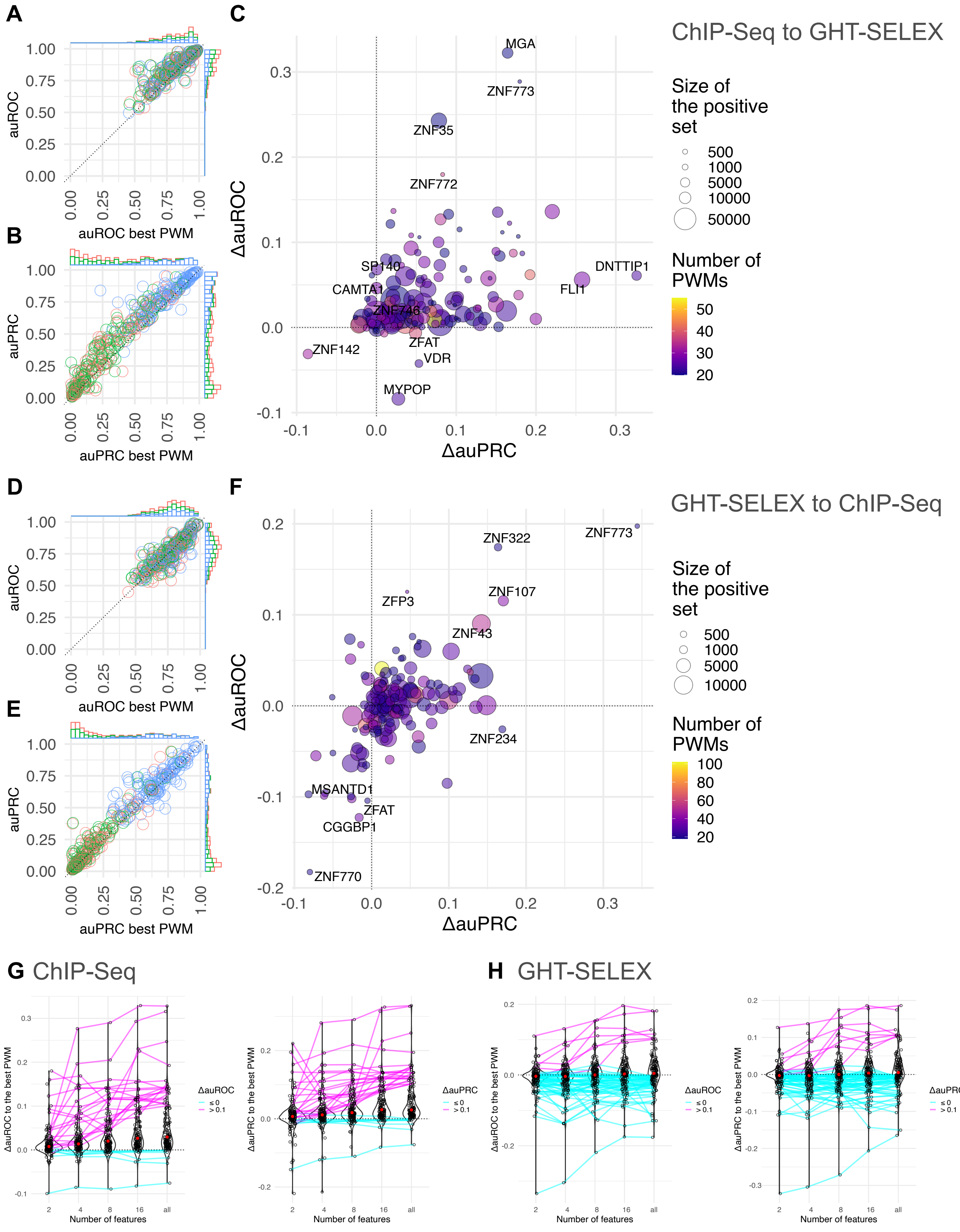

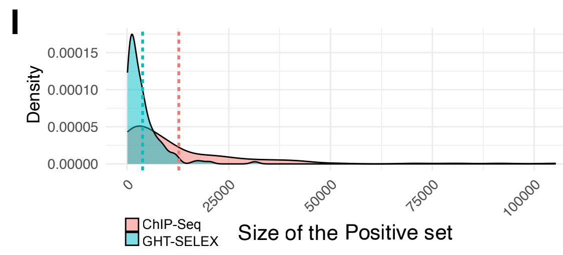

Supplementary Figure 10. Performance of the Archipelago model when trained on ChIP-Seq and tested on GHT-SELEX and vice versa. **A-C:** Same as in Supplementary Figure 8 A-C but for the model trained on ChIP-Seq and tested on GHT-SELEX. **D-F:** Same as **A-C** but for GHT-SELEX-to-ChIP-Seq transfer. Estimates obtained with alternative negative sets (random, aliens, and shades) are depicted by colored circles (green, red, and blue, respectively). **G, H:** Performance gain (purple) or loss (blue) depending on the number of PWMs in the model, ChIP-Seq-to-GHT-SELEX and GHT-SELEX-to-ChIP-Seq. **I:** Training data set size for ChIP-Seq and GHT-SELEX.

Codebook/GRECO-BIT Motif Explorer Website Overview

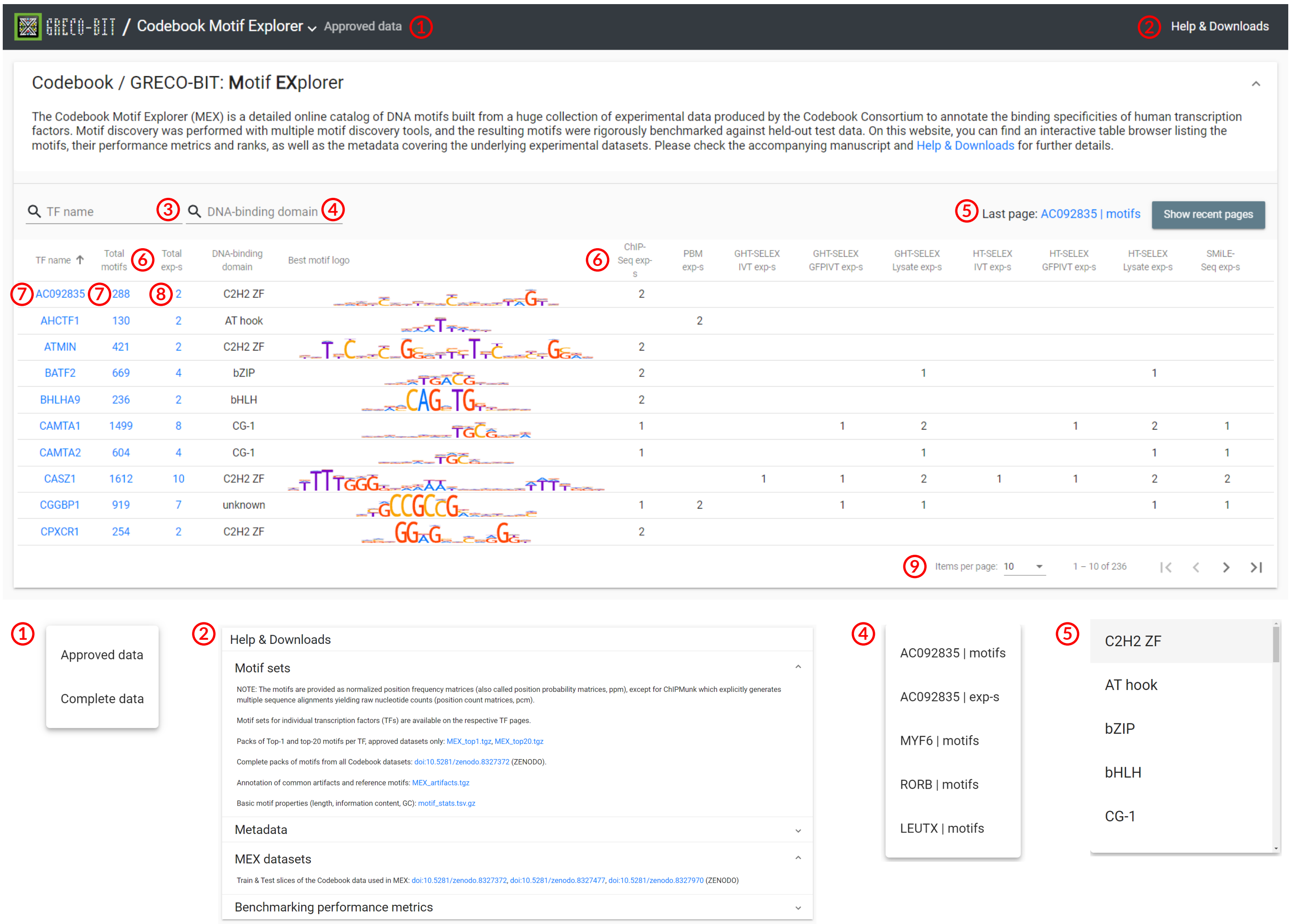

① Swtich between **Approved** and **Complete** sets of motifs and experiments

② Link to the brief **Help** and **Downloads** page

③ Filter the list of TFs and representative motifs by **TF name** or ④ **DNA-binding domain**

⑤ Quick **Page navigation** menu

⑥ Sort the table by clicking on **column headers** (such as the number of experiments a particular type)

⑦ Link to the page listing the **top-ranked motifs** of a particular **TF**

⑧ Link to the page with the **experiment-level metadata**

⑨ Table **pagination** settings

Supplementary Figure 11.
An overview of features of the Codebook Motif Explorer website (https://mex.autosome.org).

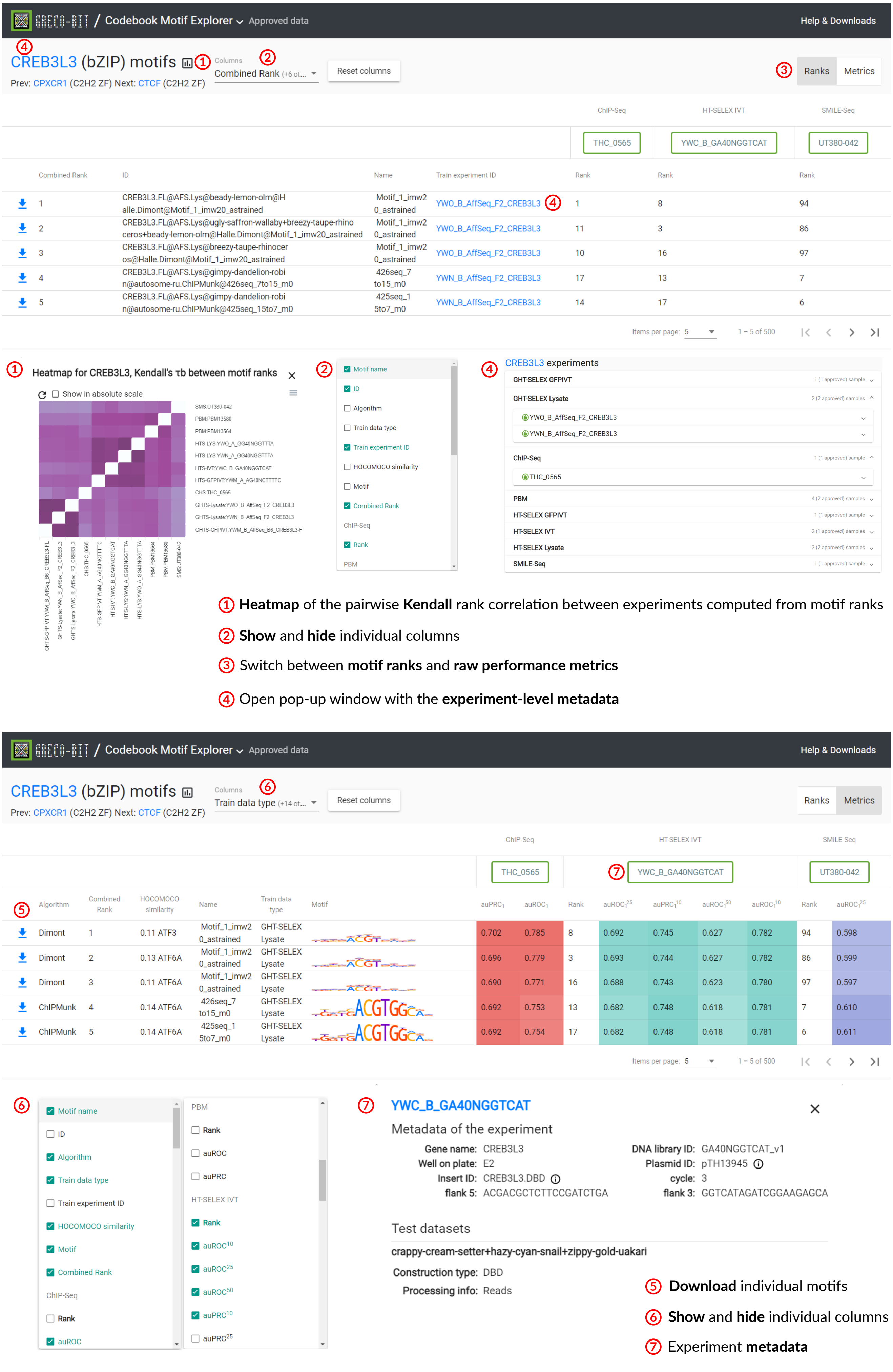

Supplementary Figure 11, cont.

An overview of features of the Codebook Motif Explorer website (https://mex.autosome.org).

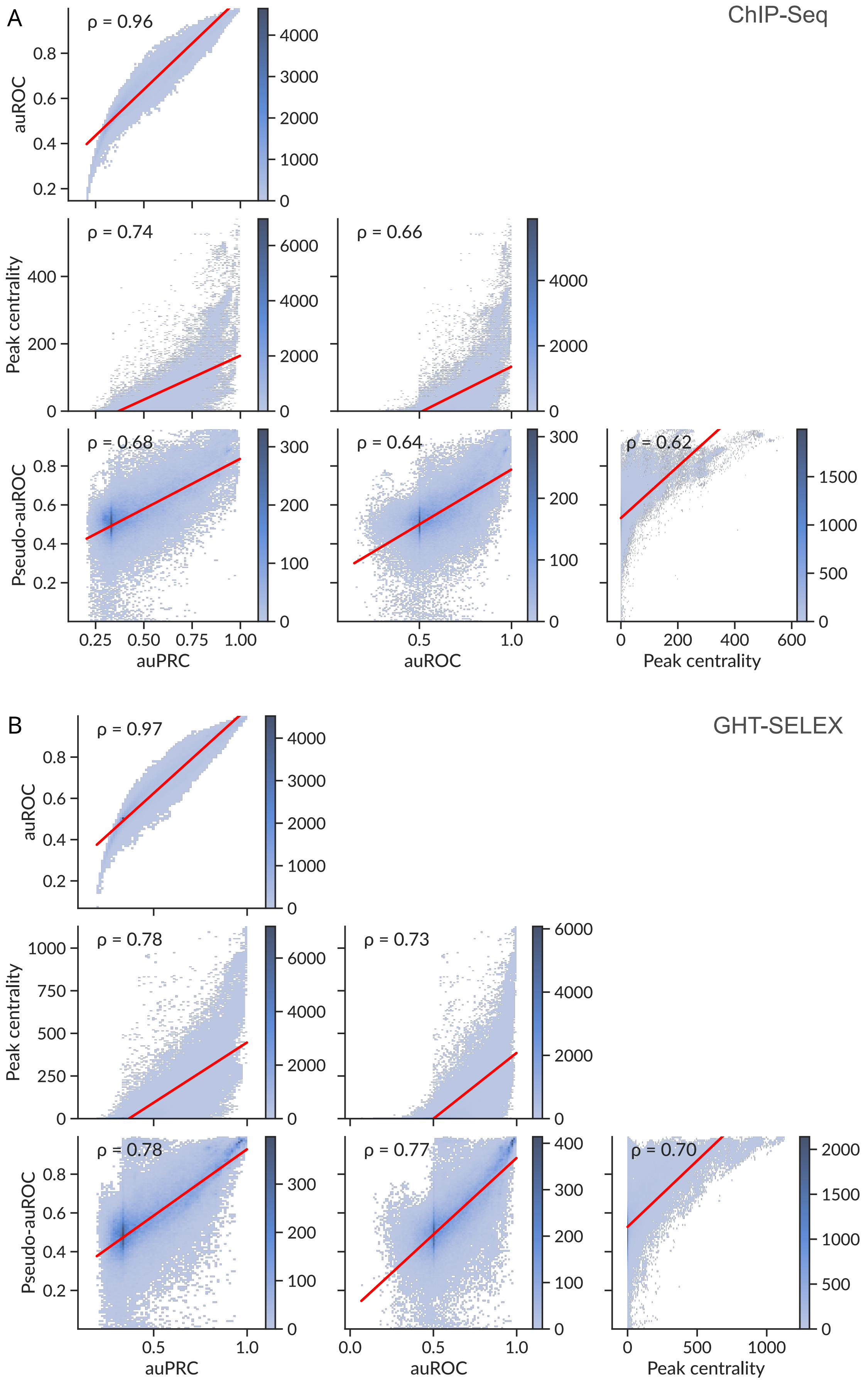

Supplementary Figure 12. Correlations of different benchmarking metrics considering all pairs of motifs and datasets. Linear regression trend (red line) and Pearson's rho are shown on the plots. **A:** ChIP-Seq. **B:** GHT-SELEX.
The color scale of the binned density plots denotes the number of motifs.
