# Supplementary material for: Cross-platform motif discovery and benchmarking to explore binding specificities of poorly studied human transcription factors
Source: Commun Biol. 2025 Nov 7;8:1545. doi: 10.1038/s42003-025-08909-9 (PMC12594988; doi:10.1038/s42003-025-08909-9)
Supplement: Supplementary file 3 — Description of Additional Supplementary files [file 42003_2025_8909_MOESM3_ESM.pdf]

Description of Additional Supplementary files

File name: Supplementary Data 1

Description: Overview of the Codebook experiments used in the study.

File name: Supplementary Data 2

Description: Analysis of the transferability of Archipelago models.

File name: Supplementary Data 3

Description: List of software tools used in the study.
