# Supplementary material for: Cross-platform motif discovery and benchmarking to explore binding specificities of poorly studied human transcription factors
Source: Commun Biol. 2025 Nov 7;8:1545. doi: 10.1038/s42003-025-08909-9 (PMC12594988; doi:10.1038/s42003-025-08909-9)
Supplement: Supplementary file 7 — Reporting Summary [file 42003_2025_8909_MOESM7_ESM.pdf]

nature portfolio

Corresponding author(s): Vsevolod J. Makeev, Timothy R. Hughes, Ivan V. Kulakovskiy

Last updated by author(s): Aug 29, 2025

Reporting Summary

Nature Portfolio wishes to improve the reproducibility of the work that we publish. This form provides structure for consistency and transparency in reporting. For further information on Nature Portfolio policies, see our Editorial Policies and the Editorial Policy Checklist.

Statistics

For all statistical analyses, confirm that the following items are present in the figure legend, table legend, main text, or Methods section.

n/a	Confirmed	
☐	☒	The exact sample size (*n*) for each experimental group/condition, given as a discrete number and unit of measurement
☒	☐	A statement on whether measurements were taken from distinct samples or whether the same sample was measured repeatedly
☐	☒	The statistical test(s) used AND whether they are one- or two-sided<br>*Only common tests should be described solely by name; describe more complex techniques in the Methods section.*
☐	☒	A description of all covariates tested
☐	☒	A description of any assumptions or corrections, such as tests of normality and adjustment for multiple comparisons
☐	☒	A full description of the statistical parameters including central tendency (e.g. means) or other basic estimates (e.g. regression coefficient) AND variation (e.g. standard deviation) or associated estimates of uncertainty (e.g. confidence intervals)
☒	☐	For null hypothesis testing, the test statistic (e.g. *F*, *t*, *r*) with confidence intervals, effect sizes, degrees of freedom and *P* value noted<br>*Give P values as exact values whenever suitable.*
☒	☐	For Bayesian analysis, information on the choice of priors and Markov chain Monte Carlo settings
☒	☐	For hierarchical and complex designs, identification of the appropriate level for tests and full reporting of outcomes
☐	☒	Estimates of effect sizes (e.g. Cohen's *d*, Pearson's *r*), indicating how they were calculated

Our web collection on statistics for biologists contains articles on many of the points above.

Software and code

Policy information about availability of computer code

Data collection	The experimental data underlying this study are described in the accompanying manuscript (Jolma et al., 2024, 10.1101/2024.11.11.622097). The comprehensive list of analyzed datasets is provided as Supplementary Data 1.
Data analysis	The implementation of the data processing pipeline is available on GitHub (https://github.com/autosome-ru/greco-bit-data-processing).<br>The motif benchmarking protocols are available on GitHub (https://github.com/autosome-ru/motif_benchmarks).<br>The comprehensive list of software tools used in the study is provided in Supplementary Data 3.

For manuscripts utilizing custom algorithms or software that are central to the research but not yet described in published literature, software must be made available to editors and reviewers. We strongly encourage code deposition in a community repository (e.g. GitHub). See the Nature Portfolio guidelines for submitting code & software for further information.

Data

Policy information about availability of data

All manuscripts must include a data availability statement. This statement should provide the following information, where applicable:

- Accession codes, unique identifiers, or web links for publicly available datasets
- A description of any restrictions on data availability
- For clinical datasets or third party data, please ensure that the statement adheres to our policy

The interactive Codebook/GRECO-BIT Motif Explorer website is available online at https://mex.autosome.org. The complete set of MEX motifs and the

benchmarking-ready Codebook data are available on ZENODO. The Archipelago preprocessed data and trained models are available on ZENODO. The numerical source data used for plotting the Figures are available at GitHub: https://github.com/autosome-ru/TSV_data_for_Codebook_MEX_figures and https://github.com/autosome-ru/MEX-ArChIPelago.

Research involving human participants, their data, or biological material

Policy information about studies with human participants or human data. See also policy information about sex, gender (identity/presentation), and sexual orientation and race, ethnicity and racism.

Reporting on sex and gender	Our study did not involve human participants.
Reporting on race, ethnicity, or other socially relevant groupings	NA
Population characteristics	NA
Recruitment	NA
Ethics oversight	NA

Note that full information on the approval of the study protocol must also be provided in the manuscript.

Field-specific reporting

Please select the one below that is the best fit for your research. If you are not sure, read the appropriate sections before making your selection.

☒ Life sciences ☐ Behavioural & social sciences ☐ Ecological, evolutionary & environmental sciences

For a reference copy of the document with all sections, see nature.com/documents/nr-reporting-summary-flat.pdf

Life sciences study design

All studies must disclose on these points even when the disclosure is negative.

Sample size	No sample-size calculation was performed.
Data exclusions	Our analysis involved an extensive human expert curation of the Codebook datasets (described in Jolma et al., 2024, 10.1101/2024.11.11.622097). Only curation-approved datasets were used for the in-depth analysis. The curation results are provided in Supplementary Table ST1. The curation approach is described in Methods.
Replication	Multiple replicates were analyzed independently, when available. Reliability of the experimental findings was ensured by the joint analyzing of results from alternative experimental assays.
Randomization	Not relevant to the design of the study as the samples were allocated into experimental groups in a deterministic way depending on the employed experimental assay.
Blinding	Blinding was not performed as we considered the complete collection of the Codebook data and applied an automated computational benchmarking procedure. The sample metadata were provided explicitly to the investigators.

Reporting for specific materials, systems and methods

We require information from authors about some types of materials, experimental systems and methods used in many studies. Here, indicate whether each material, system or method listed is relevant to your study. If you are not sure if a list item applies to your research, read the appropriate section before selecting a response.

Materials & experimental systems

n/a	Involved in the study	
☒	☐	Antibodies
☒	☐	Eukaryotic cell lines
☒	☐	Palaeontology and archaeology
☒	☐	Animals and other organisms
☒	☐	Clinical data
☒	☐	Dual use research of concern
☒	☐	Plants

Methods

n/a	Involved in the study	
☒	☐	ChIP-seq
☒	☐	Flow cytometry
☒	☐	MRI-based neuroimaging

Plants

Seed stocks	NA
Novel plant genotypes	NA
Authentication	NA
